# 2-(3-Hydroxy-5-phosphonooxymethyl-2-methyl-4-pyridyl)-1,3-thiazolidine-4-carboxylic Acid, Novel Metabolite of Pyridoxal 5′-Phosphate and Cysteine Is Present in Human Plasma—Chromatographic Investigations

**DOI:** 10.3390/ijms21103548

**Published:** 2020-05-18

**Authors:** Justyna Piechocka, Monika Wrońska, Iwona E. Głowacka, Rafał Głowacki

**Affiliations:** 1 Department of Environmental Chemistry, Faculty of Chemistry, University of Lodz, 163 Pomorska Str., 90-236 Łódź, Poland; m.wronska17@onet.eu; 2 Laboratory of Bioorganic Chemistry, Faculty of Pharmacy, Medical University of Lodz, 1 Muszyńskiego Str., 90-151 Łódź, Poland; iwona.glowacka@umed.lodz.pl

**Keywords:** cysteine, gas chromatography–mass spectrometry, human plasma, pyridoxal 5′-phosphate, N-trimethylsilyl-N-methyl trifluoroacetamide, 2-(3-hydroxy-5-phosphonooxymethyl-2-methyl-4-pyridyl)-1,3-thiazolidine-4-carboxylic acid

## Abstract

It is well-established that aminothiols, to which cysteine (Cys) belongs, are highly reactive towards aldehydes in an aqueous environment, forming substituted thiazolidine carboxylic acids. This report provides evidence that formation of the product containing a thiazolidine ring through non-enzymatic condensation of Cys and an active form of vitamin B6 pyridoxal 5′-phosphate (PLP) occurs in vivo in humans. To prove this point, a new method, based on a gas chromatography coupled with mass spectrometry (GC-MS), has been designed to identify and quantify Cys and PLP adduct, 2-(3-hydroxy-5-phosphonooxymethyl-2-methyl-4-pyridyl)-1,3-thiazolidine-4-carboxylic acid (HPPTCA) in human plasma. The GC-MS assay relies on sample deproteinization by ultrafiltration over cut-off membranes and preconcentration by drying under vacuum, followed by treatment of the residue with derivatization mixture containing anhydrous pyridine, N-trimethylsilyl-N-methyl trifluoroacetamide (MSTFA) and trimethylchlorosilane (TMCS). The method quantifies HPPTCA in a linear range from 1 to 20 µmol L^−1^, where the lowest standard on the calibration curve refers to the limit of quantification (LOQ). The validity of the method was demonstrated. Furthermore, the method was successfully applied to plasma samples donated by apparently healthy volunteers and breast cancer patients. The GC-MS assay provides a new tool that will hopefully facilitate studies on the role of HPPTCA in living systems.

## 1. Introduction

The association between sulfur-containing amino acids and origination of several civilization diseases is well-documented. In particular, the imbalance in physiological levels of cysteine (Cys) and its homologue homocysteine (Hcy) precedes the development of some types of cancer, as well as neurodegenerative and cardiovascular diseases, while the rise in plasma Hcy levels is considered to be the risk predictor of mentioned disorders [1,2,3,4,5,6,7,8]. On the other hand, clinical trials show that high-risk patients do benefit from the lowering of plasma Hcy by vitamin B supplementation, including vitamin B6 [4,5,6,7,8]. Unfortunately, the reasons for this beneficial impact still remain ambiguous.

Vitamin B6 is a generic term for a group of three related water-soluble compounds, namely pyridoxine (PN), pyridoxal (PL) and pyridoxamine, and their phosphorylated derivatives. Three major forms, namely pyridoxal 5′-phosphate (PLP), PL and 4-pyridoxic acid are present in all human plasma samples [9]. Among them, PLP is known to be the most active form of vitamin B6 in humans. It serves as a cofactor for enzymes involved in a wide range of biochemical reactions required for normal functioning of many biological systems within the body [10,11]. Particularly, PLP acts as an essential coenzyme for cystathionine β-synthase and cystathionine γ-lyase, enzymes of the transsulfuration pathway responsible for conversion of Hcy to Cys. This irreversible process is generally favorable from the standpoint of amino acid metabolic flows in animals and humans. In this way, each factor contributing to PLP depletion might be detrimental to living organisms.

Interestingly, PLP is also known to undergo non-enzymatic condensation with amino acids, such as Cys and Hcy [12,13,14,15,16,17,18,19,20,21]. It has been reported that interaction between them results in facile formation of substituted thiazolidine [12,13,14,15,16,20,21] or thiazine [12,13,17,18,19,21] carboxylic acids, respectively. So far, the reversible equilibrium between Cys/Hcy and PLP, representing an interaction between the formyl group of PLP and both sulfhydryl and amino groups of mentioned aminothiols, has been studied repeatedly. In general, it has been found that a relatively stable product containing a thiazolidine ring is formed when these functional groups are in suitable proximity. Moreover, it has long been known that the complex formation between PLP and Cys is based on Schiff reactions [13,14,15,16] occurring in aqueous media over a wide pH range 4–10 [13,14,15,21], in particular under physiological conditions. The series of experiments have also shown that the speed of the reaction at given temperature is significantly affected by pH [13,14,21]. In recent years, it has been also reported that the Cys and PLP adduct is easily formed in the presence of rat liver supernatant [20] and human plasma [21], which did not accelerate the rate of the condensation reaction. As such condensation reactions occur in vitro and substrates, namely Cys/Hcy and PLP, are ubiquitous in human system, one would expect that corresponding derivatives would also be present. However, it is not known whether PLP- and Cys/Hcy-derived metabolites are present in vivo as yet. To the best of our knowledge, the literature is devoid of such data. 

In the light of the above-mentioned information, we have decided to take on the challenge of approaching this problem qualitatively and quantitatively. Currently, blood (plasma) and urine are gold standards in the fields of clinical, toxicological and forensic science. Plasma was the matrix of our first choice, as its composition remains constant regardless of random variables and contains appropriate substrates for particular thiazolidine and thiazine carboxylic acids. Since the concentration of plasma Cys is about 100 times as high as Hcy [9,21,22], we have predominantly attributed our efforts to 2-(3-hydroxy-5-phosphonooxymethyl-2-methyl-4-pyridyl)-1,3-thiazolidine-4-carboxylic acid (HPPTCA). Thus, the aims of the research comprised five principal steps: (**1**) synthesis, purification and confirmation of HPPTCA chemical structure; (**2**) reinvestigation of the reactivity of Cys towards PLP in vitro and in vivo; (**3**) determination of the stability of the analyte and standard under real storage conditions; (**4**) development of a highly effective analytical tool based on gas chromatography coupled with mass spectrometry (GC-MS) for identification and quantification of HPPTCA in human plasma; (**5**) application of the assay to real samples in order to confirm or exclude the presence of HPPTCA in humans. We hope that the article will contribute to further interest in the field to be discussed.

## 2. Results and Discussion 

It is well-known that a successful analysis of a particular sample still cannot be achieved without an appropriate, convenient and reliable methodology. For assays based on separation techniques, optimizing both sample preparation and separation conditions require careful consideration during a new method development. Thus, detailed experiments were carried out in order to provide reliability of the data from the newly developed methodology. 

### 2.1. Sample Preparation 

In the present study, sample preparation protocol included plasma deproteinization by ultrafiltration over cut-off membranes and preconcentration by drying under vacuum, followed by treatment of the residue with derivatization mixture containing anhydrous pyridine, N-trimethylsilyl-N-methyl trifluoroacetamide (MSTFA) and trimethylchlorosilane (TMCS). Experiments concerning optimization of sample deproteinization and analyte preconcentration were conducted with the use of hydrophilic interaction liquid chromatography (HILIC) with spectrophotometric detection (UV). Our earlier experiments have shown that HILIC coupled with beneficial UV properties of HPPTCA enables its convenient monitoring, provided that relatively high concentrations are studied [21]. Notably, this approach produced meaningful results and was particularly advantageous from the standpoint of workflow simplification and interpretation of results upon completion of optimizing protein removal and preconcentration steps. Afterwards, experiments were performed using procedures described herein (see Section 3.6 and Section 3.7). In the present study, we decided to employ GC-MS, as a more powerful tool for plasma analysis was needed. As previously reported, although the HPLC-UV assay makes direct analysis of samples possible, the method is not sensitive enough to determine HPPTCA in normal human plasma. Moreover, the convincing reason for using GC-MS was that this technique is generally characterized by high-throughput potential, sensitivity, specificity and great resolution, along with higher degrees of reproducibility and accuracy than HPLC-UV. 

#### 2.1.1. Proteins Removal 

Human plasma consists of almost 90% water, while the remaining part is made up primarily of proteins which may attenuate the performance of the analytical procedure [23]. In the present study, proteins were removed by simple ultrafiltration over a 10 kDa cut-off membrane that neither changed the pH nor caused analyte dilution. For sample deproteinization, centrifugal concentrators with polyethersulfone (PES) membrane were exploited. Since the recovery of analyte from the filters was found to be 95% and high flow rates were achieved, no additional work was undertaken to further optimize pore size or membrane material. Importantly, we found that HPPTCA is bound to proteins, as measured concentrations were lower and markedly differed from those apparent on standard solution analysis. Hence, further investigations were conducted in order to increase HPPTCA recovery from plasma proteins. 

**Figure 1:**
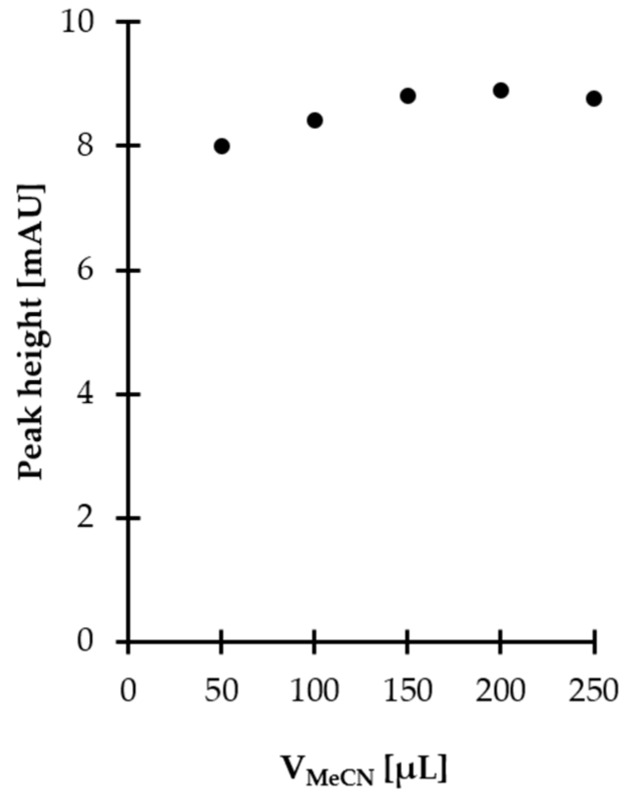
Influence of MeCN amount on HPPTCA release from plasma proteins, expressed as a peak height of HPPTCA. Samples were analyzed according to a previously published HPLC-UV assay [21].

Very promising results were obtained when inorganic acids such as perchloric acid (PCA) and trichloroacetic acid (TCA) were used. It was found that sample acidification increased recovery of the analyte up to 100%, indicating that HPPTCA is bound to proteins in a non-covalent way and can be released from denatured proteins. Unfortunately, this approach had practical limitations, as it was concomitant with a decrease in reactivity of the target derivatization agent toward HPPTCA. Moreover, it affected stability of HPPTCA, as previously reported [20,21]. In the next step, an approach utilizing organic solvent addition for protein removal was tested. Several organic solvents, including acetonitrile (MeCN), ethanol (EtOH), methanol (MeOH), 2-propanol (i-PrOH) and acetone, were used, revealing recoveries of HPPTCA reaching 25%–40%. MeCN was found to be superior with regard to extraction efficiency. Additional experiments were conducted to establish the optimal amount of MeCN and extraction time. Five different volumes, 50, 100, 150, 200 and 250 µL, were tested, while an excess addition of MeCN was excluded due to concomitant analyte dilution. The most satisfactory results were obtained when 150 µL of MeCN was used (Figure 1). It was also found that extraction time, tested in the range of 0–30 min, did not significantly affect its efficiency. Finally, 10 min extraction time was chosen, as this was considered well within the timeframe required for pretreatment of ultrafiltration concentrators and processing of samples. In summary, for routine analysis, 250 µL of plasma was mixed with 150 µL of MeCN, vigorously shaken for 10 min and then filtered on cut-off membranes. Under these conditions, about 40% HPPTCA was found to be present in the obtained filtrate taken for the next processing steps. Importantly, such recovery was reproducible regardless of HPPTCA concentration. In order to establish the effect of the analyte concentration on the recovery of HPPTCA from plasma proteins, we approached this problem quantitatively by triplicate analysis of plasma samples spiked with HPPTCA to provide final concentrations of 20 and 100 µmol L^−1^. We found that the extraction efficiency did not deviate by more than 0.5%, regardless of analyte concentration. 

#### 2.1.2. Preconcentration

**Figure 2:**
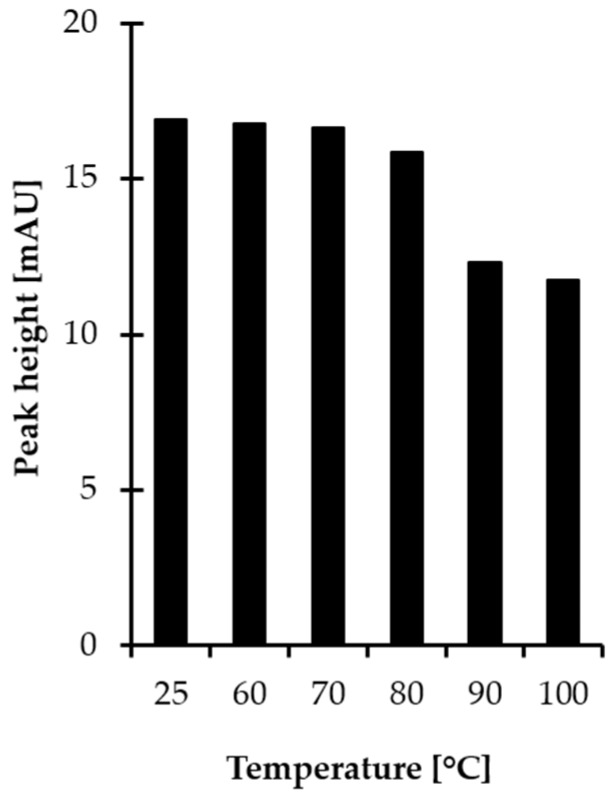
Influence of temperature on HPPTCA stability, expressed as a peak height of HPPTCA. Samples were assayed according to a previously published procedure based on HPLC-UV measurements [21].

Most derivatization reactions and reagents for gas chromatography (GC) are sensitive to water, which may slow or completely stop the reaction and/or decompose the reagent [24,25,26]. Since the target GC derivatization agent, MSTFA, is sensitive to moisture [24], the procedure encompassed a preconcentration step before chemical modification in order to remove solvents from sample. In the present study, filtrates obtained after deproteinization were dried under vacuum at elevated temperature. In order to establish optimal conditions, additional experiments were performed by setting the temperature to various values. As a result, it was found that HPPTCA is sensitive to temperature since the progressive reduction of the signal peak area was observed concomitant with temperature rise from 60 to 100 °C (Figure 2). Finally, the centrifugal vacuum concentrator was kept at the temperature of 80 °C as a compromise between reduction in drying time to 20 min and stability of HPPTCA. Under these conditions, approximately 5% of the analyte was decomposed to its substrates. 

#### 2.1.3. Derivatization 

**Figure 3:**
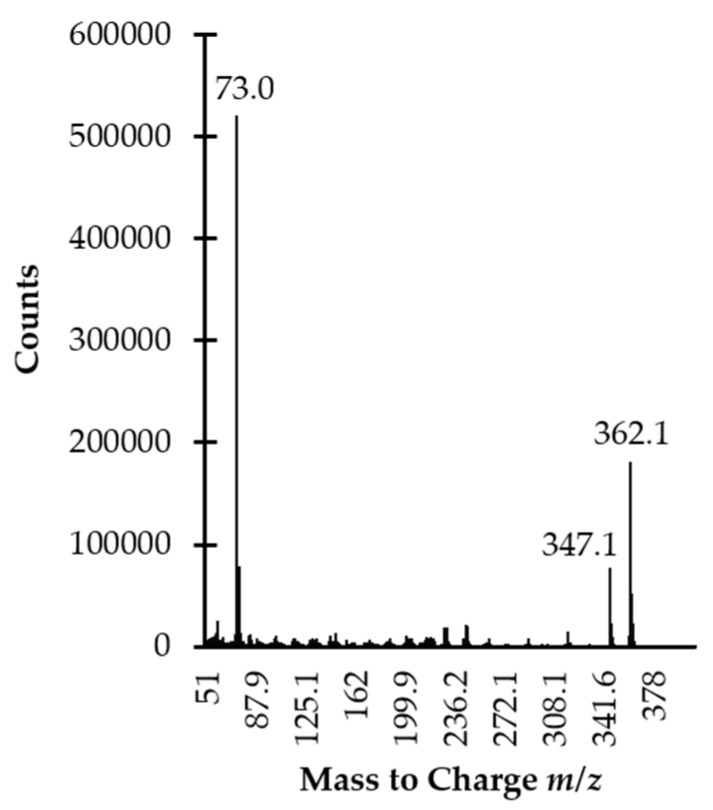
Electron ionization scan mode mass spectra of the HPPTCA-TMS derivative obtained by analyzing standard solution of HPPTCA (100 µmol L^−1^) prepared according to the procedure described in Section 3.6.

As it was envisioned, HPPTCA was difficult to analyze by GC as it was not sufficiently volatile. Therefore, a chemical modification step was employed. For this study, the use of silylation reagent MSTFA purchased as a solution containing 1% TMCS was evaluated. Silylation is one of the most widely used methods for enhancing the volatility of organic analytes in GC. Under anhydrous conditions, reactivity of functional groups towards silylation is as follows: alcohols > phenols > acids > amines > amides [27]. It is also well-established that other moieties, such as phosphate groups, can be easily silylated [28]. First, it has been indicated that upon derivatization HPPTCA is converted into trimethylsilyl-HPPTCA derivative (HPPTCA-TMS). Moreover, it was found that silylation was chiefly valuable since the HPPTCA-TMS derivative produces specific ions that are suitable for analyte monitoring (Figure 3), while the introduction of the TMS group increases the volatility of the analyte, improving GC–mass spectrometry (MS) performance. 

**Figure 4:**
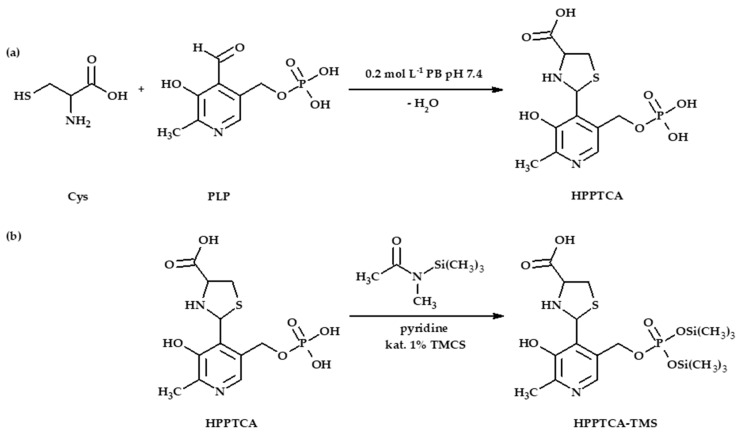
(**a**) The reaction equation of Cys with PLP providing HPPTCA. (**b**) Chemical derivatization reaction of HPPTCA with MSTFA in the presence of TMCS, affording HPPTCA-TMS derivative.

HPPTCA possesses five potential places accessible to silylation, provided by phenol, carboxyl, amine and phosphate groups. In addition, HPPTCA-TMS fragmentation ions (Figure 3) have indicated that the hydroxyl group in the pyridine ring is not silylated under experimental conditions. Taking into account reactivity of other groups, we can reasonably suppose that the carboxyl and amino groups in the thiazolidine ring remain unmodified as well. Importantly, such circumstances did not affect effective separation and detection of HPPTCA. Thus, the most probable schematic of silylation reaction of HPPTCA, formed in the reaction of PLP with Cys (Figure 4a), can be shown as proposed in Figure 4b. 

**Figure 5:**
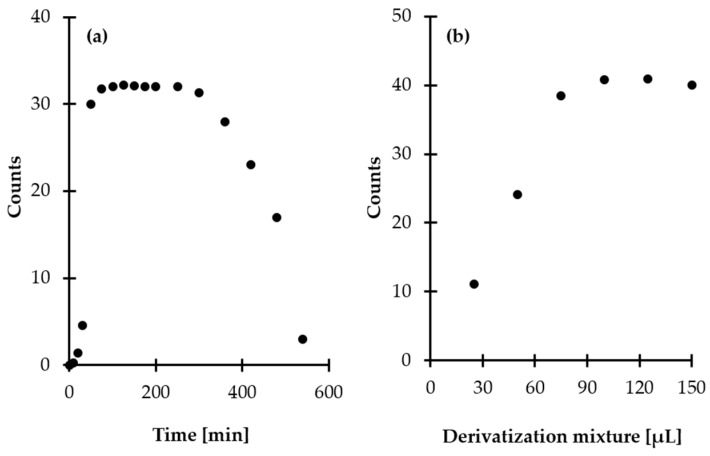
Derivatization reaction yield as a function of (**a**) reagent excess and (**b**) time combined with examination of HPPTCA-TMS stability in autosampler, expressed as a peak area of HPPTCA-TMS derivative. Samples were analyzed according to the procedure described in Section 3.7.

Then, experiments were conducted to establish the optimal amount of derivatization mixture. Based on our previous findings [29], freshly prepared derivatization mixture was used, where the ratio of pyridine to MSTFA with 1% TMCS was 1:1. It was found that the best results were obtained when the residue of plasma filtrate was treated with 100 µL of derivatization mixture (Figure 5a). Moreover, it has been noticed that temperature of the reaction, tested in the range of 25–50 °C, did not significantly affect the efficiency of the reaction. 

Further experiments were performed to establish the derivatization reaction kinetics at room temperature. Notably, it was found that the HPPTCA-TMS derivative signal reaches its maximum in 100 min after mixing of the reagents, and it remains stable for at least 3 h under experimental conditions. Then, a progressive decrease of the signal peak area was observed (Figure 5b). Since silyl derivatives tend to be moisture-sensitive [24], this phenomenon was not surprising but should be taken into account in any attempt to measure HPPTCA content using the proposed GC-MS assay. 

These experiments have established an optimal procedure, in which deproteinized plasma is dried under vacuum, treated with freshly prepared MSTFA and TMCS mixture in pyridine and subjected to the GC system after completion of the derivatization reaction. The overall sample preparation time was estimated to be 140 min. 

### 2.2. Stability of HPPTCA

Chemical compounds can be decomposed prior to chromatographic investigations under different circumstances. Therefore, the analyte and standards were evaluated in terms of their stability during method development. In particular, we approached this problem qualitatively in order to observe potential conversion of HPPTCA to Cys and PLP and quantitatively for the purpose of measuring of the intactness the analyte in a given matrix under specific storage and use conditions for preselected time intervals. In the stability experiments, samples were assayed by methods developed in our earlier studies [21,22]. Cys was quantified by a method using high-performance liquid chromatography (HPLC) with pre-column derivatization with 2-chloro-1-methyllepidinium tetrafluoroborate (CMLT) and UV detection [22], while a method based on HILIC separation that exploits UV absorption properties of HPPTCA and PLP was used for their simultaneous monitoring [21].

**Figure 6:**
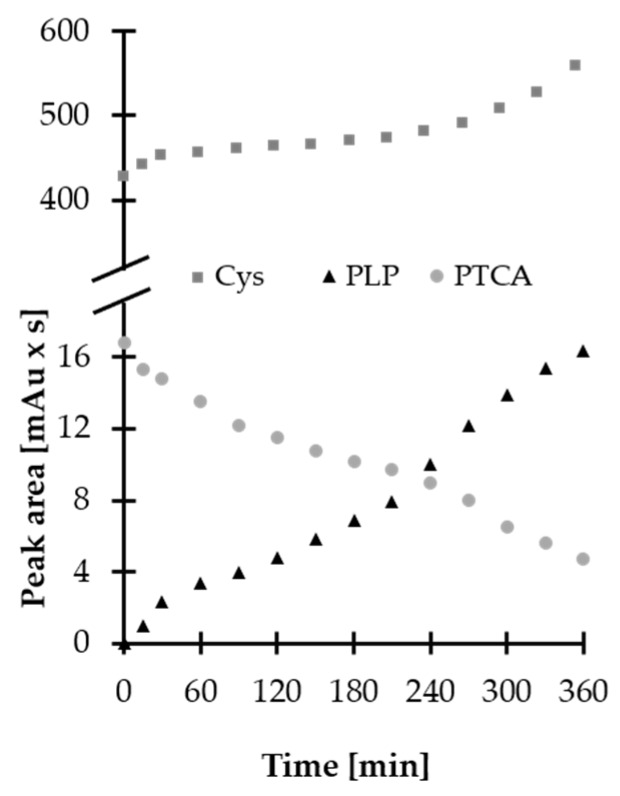
Stability of HPPTCA in human plasma spiked with the analyte (100 µmol L^−1^) at room temperature, expressed as a peak area. Samples were processed according to previously published methods based on HPLC-UV [21,22].

In the beginning, short-term stability of stock solution of HPPTCA was evaluated. Since HPPTCA was found to be soluble in water solvents only, the HPPTCA powder was dissolved in deionized water to provide final concentrations of 100 and 1000 µmol L^−1^. In addition, standard solutions of HPPTCA (100 µmol L^−1^) were prepared in 0.2 mol L^−1^ phosphate buffer (PB), and different pH values (4.0, 5.0, 6.0, 7.4 and 8.0) were tested, covering the expected pH of human biofluids such as plasma, urine and saliva. The stability of stock solutions was evaluated at room temperature for 6 or 48 h, where standard solutions of HPPTCA at 100 and 1000 µmol L^−1^ concentration, respectively, were analyzed. Importantly, it was recognized that the standard solution of HPPTCA is unstable under experimental conditions. Moreover, it was noticed that the process of degradation occurs more slowly in acidic solutions rather than alkaline, while cooling of the sample is only able to delay this effect but does not eliminate it. Notably, it was found that HPPTCA is gradually decomposed to its substrates. Interestingly, the same phenomenon was observed when plasma samples spiked with HPPTCA ( 100 µmol L^−1^) were assayed. As shown in Figure 6, the decrease in peak area of HPPTCA was concomitant with the increase in concentration of Cys and PLP in the subjected sample, indicating that condensation of the aminothiol and aldehyde is reversible, particularly under slightly alkaline conditions [20]. Short-term stability of HPPTCA in normal plasma samples was not tested because of its degradation during the time-consuming sample preparation process for GC measurements. Nevertheless, native samples assayed for HPPTCA in successive days were found to be stable, provided that they were kept at −80 °C. Finally, it was found that stock solution of HPPTCA (1 mmol L^−1^) remains stable for no longer than 4 h at room temperature, as its concentration did not deviate by more than 5%. In comparison, approximately 40% of the analyte was decomposed when 100 µmol L^−1^. HPPTCA water solution was examined under experimental conditions.

Moreover, the stability of the HPPTCA powder was evaluated as a pure standard for the first time ever. Since our preliminary studies have indicated that HPPTCA is sensitive to moisture and light, the powder was stored in an airtight jar made with amber glass. This observation was in an agreement with F. Bergel and K. R. Harrap, who reported a previous unsuccessful attempt to synthesize pure HPPTCA [14]. Moreover, it was placed in an ultra-low-temperature freezer at −80 °C to prolong the stability of the obtained product. So far, experiments have shown that solid HPPTCA kept under recommended storage conditions is stable for at least five months. Nevertheless, these studies are still ongoing.

Finally, it needs to be emphasized that these studies have provided only preliminary, but still valuable, data on the stability of HPPTCA, indicating new directions in the field. In particular, they have shown that due to the short residence time of the analyte in plasma, it would be reasonable to establish a stabilizer to be used during sample collection and handling. On the other hand, these studies have indicated that HPPTCA might act as a reservoir of PLP and/or Cys in vivo. In our opinion, these topics remain to be reinvestigated in detail in the near future.

### 2.3. GC Separation and MS Detection

**Figure 7:**
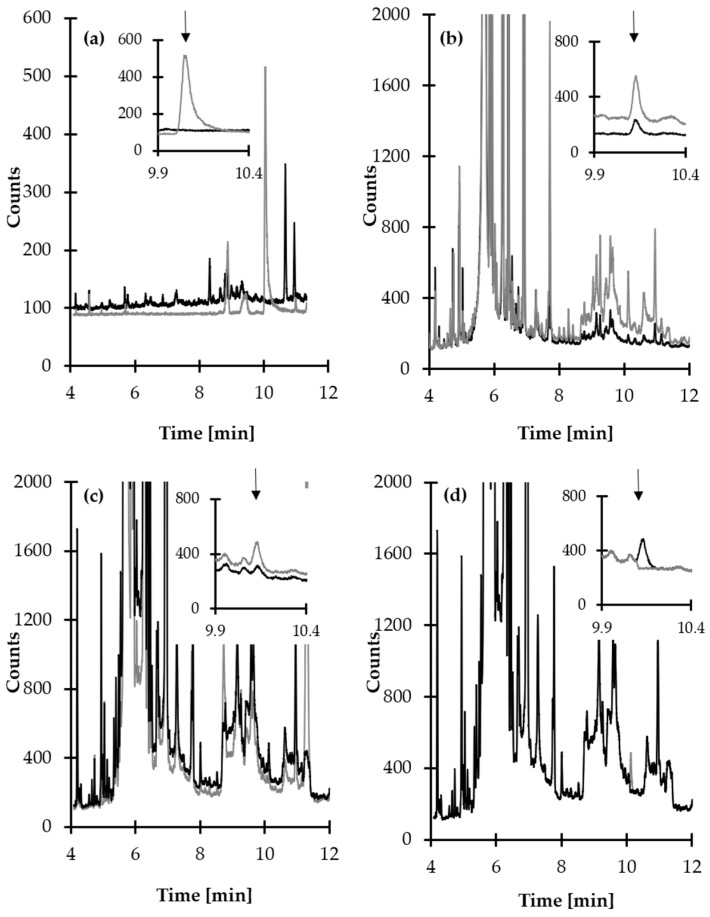
Representative chromatograms of standard solutions and human plasma prepared according to the procedure described in Section 3.6. Chromatographic conditions were as described in Section 3.7. (**a**) Blank standard solution (black line) and standard solution of HPPTCA (10 µmol L^−1^) (grey line); (**b**) normal human plasma sample (black line) and the same sample spiked with HPPTCA (10 µmol L^−1^) (grey line); (**c**) human plasma collected before (black line) and after 12 h intake of vitamin B6 (grey line); (**d**) normal human plasma sample (black line) and the same sample after alkalization (grey line).

A standard approach has been employed to specify optimal chromatographic separation conditions. The influence of many operating parameters of the GC-MS system on the method’s performance was assessed. In particular, satisfactory method selectivity was achieved through the selection of column temperature and the specific ions to be monitored by MS detector. Representative GC profiles for HPPTCA are shown in Figure 7a–d. The peak of HPPTCA-TMS derivative eluted at 10.2 min and was easy to distinguish and quantify from the responses of all concomitant matrix components under optimized conditions (see Section 3.7). Notably, a starting temperature no higher than 140 °C was necessary to maintain resolution of the HPPTCA-TMS derivative peak from other plasma constituents, while a ramp to 300 °C followed by slow cooling down was performed to ensure the column was clean before subsequent analyses.

The identification and confirmation of the target compound was performed by analyzing the standard solution of HPPTCA (100 µmol L^−1^) prepared according to the procedure described in Section 3.6. The analysis was carried out by GC-MS system. The MS detector was operated in scan mode within *m/z* 50–1000 range. Two ions, namely *m/z* 347.1 and *m/z* 362.1, were selected as a suitable for analyte monitoring (Figure 3). Further analyses were conducted with the selected ion monitoring MS mode. Identification and quantification of the compound of interest in real samples were based upon comparison of retention time and specific ions with a corresponding set of data obtained by analyzing authentic compound. Moreover, the origin of the 10.2 min peak was confirmed indirectly by analyzing the same sample after its alkalization. The typical disappearance of the peak of HPPTCA-TMS derivative could be observed when normal human plasma samples were treated with NaOH, providing additional evidence of the peak origin (Figure 7d).

### 2.4. Validation of Analytical Method

Our method was thoroughly validated on qualified instrument in order to enhance the quality of data. The selection of validation parameters and acceptance criteria was based upon United States Food and Drug Administration guidance for bioanalytical methods validation [30]. Essential parameters such as selectivity, accuracy, precision, linearity and limit of quantification (LOQ) were evaluated. Moreover, system suitability parameters such as chromatographic retention, tailing factor and number of theoretical plates were selected during method validation to determine instrument performance under optimized conditions. The system suitability tests were assessed by analyzing a standard solution of HPPTCA (10 µmol L^−1^) in 10 replicate injections. Importantly, good system suitability was shown, ensuring that the system was performing in a manner that leads to the production of accurate and reproducible data. Detailed data regarding the system suitability tests are gathered in Table 1.

Standard addition method was used for calibration of the method. The calibration curves were prepared in pooled plasma by spiking the matrix with known concentrations of the analyte. Pooled plasma made up of small pools of the specimens from all donations was produced in our laboratory. Since HPPTCA-free plasma was not available, the endogenous concentration of the analyte in a biological matrix was evaluated before calibration curve preparation by three replicate analysis. A calibration curve consisted of six samples covering the expected unknown sample concentration in the range of 1–20 µmol L^−1^, including LOQ. The calibration curves were run once per day over five subsequent working days. Least squares regression model was used to describe the concentration–response relationship. The linearity was evaluated graphically by visually inspecting a plot of the peak area as a function of the analyte concentration. The curve’s correlation coefficient (R) was monitored as well, showing that the detector response was proportional to the HPPTCA concentration within the intended quantitation range. Conducted experiments also indicated that only the signal peak area of HPPTCA-TMS derivative eluted at 10.2 min increased linearly with the growing concentration of HPPTCA. Importantly, the slope of the calibration curves obtained over five subsequent days did not deviate by more than 3.1%, indicating the procedure’s reliability. Moreover, matrix effect evaluation studies have indicated that optimized sample preparation procedure eliminates most of undesirable matrix components, since calibration curves prepared in human plasma samples from six individual sources did not markedly differ in slope.

Short-term accuracy and precision of the method were evaluated across the quantitation range by triplicate analysis of pooled plasma samples containing known amounts of the analyte. Three different concentrations were included: one close to the LOQ, one near the middle and one near the high end range of the calibration curve. For intermediate measurements, experiments were repeated on five subsequent days and compared with short-term precision and accuracy. Each time, freshly prepared quality controls were analyzed. All concentrations were tested with the use of calibration curves prepared specifically on that occasion. The precision determined at each concentration level did not exceed 14.35% of the coefficient of variation (CV), except for the case of the LOQ, where it did not exceed 10.45% of the CV. Accuracy was expressed as percent recovery. Recovery experiments were performed by comparing the analytical results of the mean measured amount with added amount of HPPTCA. It varied from 94.85% to 104.83% for both short-term and intermediate measurements. Notably, such good precision and accuracy were obtained with no outliners excluded. Accuracy was calculated with the use of the following formula:

Accuracy [%] = [(measured amount − endogenous content)/added amount] × 100.

The LOQ evaluation was completed as a part of the precision and accuracy assessment for the calibration range. LOQ was accepted as the lowest standard on the calibration curve, since the analyte response was identifiable, discrete and reproducible with the precision of 10.45% and accuracy of 104.83%. In the present study, the LOQ value amounts to 1 µmol L^−1^ and corresponds closely to the LOQ determined by signal-to-noise method. In this method, a proxy matrix (0.9% NaCl in 0.1 mol L^−1^ PB pH 7.4) was spiked with decreasing concentrations of the analyte and treated according to procedure described in Section 3.6 and Section 3.7 until the injected amount of HPPTCA resulted in a peak height 10 times greater than the baseline noise level.

Some attempts have been made to verify selectivity. Selectivity studies in particular assessed interferences that may be caused by matrix components such as Cys, cystine (Cys_2_) and PLP, as products of HPPTCA degradation. First, blank standard solution and standard solution of HPPTCA (10 µmol L^−1^) were analyzed. As shown in Figure 7a, the elution profile is free of interference at the retention time of the analyte. Furthermore, normal human plasma samples from six individual sources and the same specimens spiked with Cys, Cys_2_ and PLP were assayed according to the procedures described herein (see Section 3.6 and Section 3.7). In all cases, no increase in peak area of HPPTCA was observed. The analyte peak was also evaluated for purity. An MS detector operated in scan mode was used for chromatographic selectivity assessment. It was set to acquire spectra on-line throughout the entire chromatogram, and the spectra obtained during the elution of the peak were compared. The study indicated peak purity, as the same MS spectra acquired in different sections of the peak at 10.2 min were observed. An additional experiment was also performed to verify that the substance being measured is the intended analyte. According to literature data, HPPTCA is unstable under alkaline and acidic conditions [20,21]. Thus, the same blank plasma samples were treated with 1 mol L^−1^ NaOH and re-assayed. Importantly, the peak of HPPTCA-TMS derivative disappeared, indicating identity of the analyte (Figure 7d). In the end, there were no matrix effects throughout the application of the method. It has also been demonstrated that the analytical procedure has suitable levels of precision, accuracy and linearity. Therefore, the protocol did not encompass an internal standard addition step, thus simplifying sample preparation procedure. Detailed data regarding all validation parameters are shown in Table 2.

### 2.5. Application of the Method

First, the validated method was implemented to study the reactivity of Cys toward PLP in vivo. Three apparently healthy adult volunteers were involved in the experiment (two women and one man in the 28–53 age group). The volunteers had taken a medication and donated blood at the same time. One dose of 200 mg of a commercially available pharmaceutical preparation was administered to them, which did not exceed the recommended dose per day. No additional medications were allowed. Blood samples were collected just before and after 12 h of vitamin B6 supplementation. Each time, samples were taken after 3 h fast, before which they were allowed normal food and fluid intake. Samples were handled according to procedures described in Section 3.6 and Section 3.7. In each case, HPPTCA was initially present in plasma samples, and a marked increase in concentration of analyte after 12 h intake of vitamin B6 was observed (Figure 7c). The method was also used for quantitative determination of HPPTCA in plasma samples from apparently healthy volunteers as well as cancer patients. Samples with higher concentration were diluted and re-assayed. Concentrations of plasma HPPTCA varied from 18.86 to 35.34 µmol L^−1^ and from 2.14 to 21.13 µmol L^−1^ with average values of 26.28 ± 8.36 and 14.83 ± 6.36 µmol L^−1^ for healthy donors and patients, respectively. These values for plasma HPPTCA are the first ever reported.

## 3. Materials and Methods

### 3.1. Reagents and Materials

All chemicals used throughout this study were of analytical reagent grade. D,L-Cys hydrochloride and its symmetrical disulfide (Cys_2_), PLP monohydrate, MSTFA, TMCS, tris(2-carboxyethyl)phosphine hydrochloride, TCA, trifluoroacetic acid (TFA), sodium hydroxide, HPLC-gradient grade acetone, diethyl ether and anhydrous pyridine were from Sigma-Aldrich, USA. Sodium hydrogen phosphate heptahydrate, sodium dihydrogen phosphate dihydrate, HPLC-gradient grade MeCN, i-PrOH, MeOH, EtOH, phosphoric acid, PCA and acetic acid were from J.T. Baker, the Netherlands. CMLT was prepared as previously described [22]. HPPTCA was synthesized according to method described herein. For sample deproteinization, Vivaspin 500 centrifugal concentrators with PES membrane purchased from Sartorius, Germany were exploited. Commercially available 50 mg vitamin B6 tablets, containing active substance PN hydrochloride, were used.

### 3.2. Instrumentation

An Agilent 7820A GC system equipped with automated sample injector model 7693A and MS detector 5977B (Agilent Technologies, Germany) was used for GC experiments. The GC apparatus was equipped with split/splitless inlet, working in split ratio of 10:1 mode to a 30 m × 0.25 mm HP-5MS quartz capillary column with a 0.25 µm film thickness (Agilent Technologies, Germany). Data acquisition and analysis were performed using MassHunter 5977B MSD Bundle with 7820 GC software.

HPLC analyses were carried out using an Agilent 1220 Infinity LC system equipped with a binary pump integrated with two-channel degasser, autosampler, column oven and diode-array detector (DAD) (Agilent Technologies, Germany) controlled by OpenLAB CDS ChemStation software. Analytes were separated on Aeris PEPTIDE XB-C18 (150 mm × 4.6 mm, 3.6 µm) column or Kinetex HILIC column (100 mm× 4.6 mm, 2.6 μm) from Phenomenex, USA.

The pH was measured using an HI 221 pH-meter (Hanna Instrument, USA). Samples were dried using a CentriVap Centrifugal Vacuum Concentrator (Labconco, USA). For sample shaking, Multi Speed Vortex MSV-3500 (Biosan, Latvia) was used. During the study, a Mikro 220R centrifuge with fast cool function (Hettich Zentrifugen, Germany) and a QBD2 thermostat (Grant Instruments Ltd., UK) were also exploited. Water was purified using a Milli-QRG system (Millipore, Austria). Samples were stored in ultra-low-temperature freezer (Panasonic, USA). Purification of HPPTCA was performed with the use of an HPLC preparative system from Waters (USA) equipped with a 2545 binary gradient pump and a 2998 DAD detector. Nuclear magnetic resonance spectroscopic spectra (NMR) were recorded using an Avance III 600 MHz spectrometer (Bruker, USA), while MS spectra were registered using a Nexera X2 HPLC system coupled with a 8050 triple quadrupole detector (Shimadzu, Japan).

### 3.3. Synthesis of HPPTCA

The first reference of Cys reactivity toward PLP in aqueous solution was given by Y. Matsuo [12]. In the present work, we prepared pure HPPTCA according to the procedure described below. Cys (0.6305 g, 4 mmol) and PLP (0.1061 g, 0.4 mmol) were dissolved in 1 mL 0.2 mol L^−1^ PB, pH 7.4, and allowed to react for 30 min at room temperature. The reaction product, HPPTCA, was purified by HPLC using XBridge BEH C18 OBD Prep column (19 mm × 100 mm, 5 µm) from Waters (USA). After sample loading, the column was eluted with the mobile phase, which consisted of H_2_O adjusted to pH 1.7 with TFA (A) and MeCN (B), delivered at flow rate of 15 mL min^−1^. The chromatographic separation was performed at room temperature and was accomplished using gradient elution 0–1 min 5% B, 1–4 min 5%–60% B, 4–6 min 60%–5% B. The effluent was monitored at 305 nm. Fractions containing HPPTCA, a predominant product eluting at 3.03 min, were collected, and the solvent was removed with the use of rotary evaporator. Then, the residue was crystallized from the mixture of H_2_O, MeCN and diethyl ether (1:5:10, *v/v/v*). The final product, a yellowish powder, was washed with diethyl ether and then dried under high vacuum overnight at 4 °C. The reaction efficiency was in the range of 50%–60%. Pure HPPTCA was stored in an amber glass jar protected from moisture at −80 °C.

The purity of the final product was assessed by analytical HPLC according to a previously published method, and the obtained UV spectra correlated well with those recorded earlier [21]. The chemical structure of HPPTCA was confirmed using NMR. ^1^H, ^13^C and ^31^P NMR spectra were taken in DMSO-*d_6_* at 600, 151 and 242 MHz, respectively; chemical shifts δ are given in ppm and coupling constants *J* in Hz. ^1^H NMR (600 MHz, DMSO-*d_6_*) *δ* [ppm]: 11.52 (brs, 1H, COOH), 7.93 (s, 1H, NC*H*), 5.87 (s, 1H, SC*H*N), 4.94−4.91 (m, 2H, POC*H*_a_H_b_, OH), 4.88−4.83 (m, 2H, POCH_a_*H*_b_, OH), 4.11 (t, *J* = 7.3 Hz, 1H, C*H*COOH), 3.47−4.39 (m, 2H, SC*H*_a_H_b_, NH), 3.22 (dd, *J* = 10.7 Hz, *J* = 7.3 Hz, 1H, SCH_a_*H*_b_), 2.40 (s, 3H, CH_3_); ^31^P NMR (242 MHz, DMSO-*d_6_*) *δ* [ppm]: −1.44; ^13^C NMR (151 MHz, DMSO-*d_6_*) *δ* [ppm]: 172.80 (s, C=O) 152.88, 146.84, 136.11, 131.10, 129.62, 65.38, 63.25, 62.95, 62.36 (d, *J* = 5.6 Hz COP), 35.85, 15.64. ^1^H, ^13^C and ^31^P NMR spectra are shown in Figure S1a–c. Characterization of HPPTCA was also performed by LC-MS/MS technique using triple quadrupole LC-MS system. Products with protonated molecular ion [M + H]^+^ at *m/z* 351.00 and deprotonated molecular ion [M-H]^-^ at *m/z* 349.05 were observed in positive and negative mode, respectively. The observed protonated and deprotonated molecular ions matched the theoretical average molecular mass of the HPPTCA (350.28 g mol^−1^), calculated using the standard atomic weights (Figure S2).

### 3.4. Stock Solution of HPPTCA

Stock solution of HPPTCA (1 mmol L^−1^) was prepared daily by dissolving an appropriate amount of HPPTCA powder in deionized water. Then, the solution was kept at 4 °C for no longer than 4 h. The working solutions of HPPTCA were prepared by dilution of standard solution with deionized water as needed and were processed without delay. Importantly, all solutions were protected from light.

### 3.5. Biological Samples Collection

Blood samples (about 2 mL) were collected by venipuncture into sterile tubes containing EDTA, cooled on ice and centrifuged at 5000× *g* for 15 min at room temperature within 30 min. The plasma supernatant was delivered to the laboratory in a frozen state using dry ice as a cooling agent. The plasma supernatant was stored at −80 °C until analysis. Samples were processed within three weeks according to the procedure described in Section 3.6. We studied three apparently healthy volunteers and 10 anonymous patients suffering from breast cancer belonging to an ethnically homogeneous group. Neither the apparently healthy donors nor the cancer patients were supplemented with analyte before sample collection. The study was approved by the Ethical Committee of the University of Lodz (decision identification code: 20/KBBN-UŁ/III/2019).

### 3.6. Plasma Specimen Preparation for HPPTCA Quantification by GC-MS

Plasma sample (250 µL) was mixed with 150 µL of MeCN and vigorously shaken for 10 min at 3000× *g* using Multi Speed Vortex. Then 350 µL of extraction mixture was deproteinized by ultrafiltration, using a 10 kDa cut-off membrane (12,000× *g*, 10 min, 4 °C). Obtained filtrate (100 µL) was transferred into 0.5 mL polypropylene microtube and dried under vacuum (20 min at 80 °C). Thereafter, the residue was treated with 100 µL derivatization mixture containing MSTFA with 1% TMCS in pyridine (1:1, *v/v*) without delay and incubated at room temperature for 100 min. Afterwards, the reaction mixture was transferred to a vial, and 1 µL of the sample was injected into GC-MS system and analyzed according to the procedure described in Section 3.7.

### 3.7. GC-MS Conditions

Helium (99.9999%) was used as the carrier gas with a constant flow rate of 1 mL min^−1^. The injection port temperature was set to 280 °C. The chromatographic separation of HPPTCA-TMS derivative was accomplished under thermal gradient conditions. The initial oven temperature was set to 140 °C and increased to 220 °C in steps of 15 °C min^−1^, then 5 °C min^−1^ to 250 °C and finally 15 °C min^−1^ to 300 °C. Afterwards, the oven was slowly cooled down in steps of 20 °C min^−1^. The MS detector was operated in the electron impact mode at 70 eV. The ion source temperature was set at 230 °C, the temperature of quadrupole was set at 150 °C and the MS interface was set to 250 °C. The multiple ion detector was focused on the ions of HPPTCA-TMS derivative with masses of *m/z* 347.1 and *m/z* 362.1 to identify the target compound, while *m/z* 362.1 was selected for quantification.

## 4. Conclusions

To the best of our knowledge, this is the first report dealing with the in vivo formation and presence of HPPTCA in humans. Our experiments have produced plausible evidence that HPPTCA, formed in the reaction of PLP with Cys, is present in human plasma. The most astonishing aspect of our findings is that HPPTCA appears as the most abundant form of vitamin B6 in human plasma, exceeding plasma PLP level by about 1000-fold [9]. Taking into consideration the possible decomposition of HPPTCA leading to Cys and PLP release, it would be reasonable to state that HPPTCA might act as a reservoir of PLP and/or Cys in vivo. On the other hand, one can reasonably suppose that in vivo formation of HPPTCA might be detrimental to living organisms, leading to vitamin B6 depletion in humans, since the effects of vitamin B6 deficiency are widely known [10,11]. Moreover, an assay based on GC-MS technique for assessment of HPPTCA content in human plasma has been provided. Nevertheless, it needs to be clearly emphasized that the proposed approach is not free from restrictions, thus it would be reasonable to provide a more effective one in the near future. We hope that method described herein will facilitate investigation into the role of HPPTCA in living systems. So far, only one piece of evidence showing that HPPTCA might have a bactericidal effect can be found in the literature [20]. In particular, thorough studies seem to be necessary in order to better understand the fate of sulfur-containing amino acids and related compounds under physiological and pathological conditions in living organisms. Undoubtedly, these results would be desirable from the standpoint of human well-being. Hence, the subsequent project is already well underway.

## Figures and Tables

**Table 1:** System suitability test (*n* = 10).

Acceptance Criteria	Value
CV of retention time ≤ 1%	0.04%
Assymetry 0.8–1.5	0.94
Number of theoretical plates ≥ 2000	8306

**Table 2:** Validation data (*n* = 5).

Regression Equation	R	CV Slope (%)	Linear Range (µmol L^−1^)	Intermediate Precision (%)		Intermediate Accuracy (%)		LOQ (µmol L^−1^)
				Min	Max	Min	Max	
y = 0.9321x + 2.7134	0.9984	3.1	1–20	2.58	14.35	94.85	104.83	1

## Supplementary Materials

Supplementary materials can be found at https://www.mdpi.com/1422-0067/21/10/3548/s1.

## Abbreviations

CMLT	2-chloro-1-methyllepidinium tetrafluoroborate
Cys	cysteine
Cys_2_	cystine
CV	coefficient of variation
DAD	diode-array detector
EDTA	disodium ethylenediaminetetraacetate
EtOH	ethanol
GC	gas chromatography
Hcy	homocysteine
HILIC	hydrophilic interaction chromatography
HPLC	high performance liquid chromatography
HPPTCA	2-(3-hydroxy-5-phosphonooxymethyl-2-methyl-4-pyridyl)-1,3-thiazolidine-4-carboxylic acid
i-PrOH	2-propanol
LOQ	limit of quantification
MeCN	acetonitrile
MeOH	methanol
MS	mass spectrometry
MSTFA	N-trimethylsilyl-N-methyl trifluoroacetamide
NMR	nuclear magnetic resonance spectroscopy
PB	phosphate buffer
PCA	perchloric acid
PES	polyethersulfone membrane
PL	pyridoxal
PLP	pyridoxal 5′-phosphate
PN	pyridoxine
R	correlation coefficient
TCA	trichloroacetic acid
TFA	trifluoroacetic acid
TMCS	trimethylchlorosilane
TMS	trimethylsilyl group
UV	spectrophotometric detection
