# Supplementary material for: 2-(3-Hydroxy-5-phosphonooxymethyl-2-methyl-4-pyridyl)-1,3-thiazolidine-4-carboxylic Acid, Novel Metabolite of Pyridoxal 5′-Phosphate and Cysteine Is Present in Human Plasma—Chromatographic Investigations
Source: Int J Mol Sci. 2020 May 18;21(10):3548. doi: 10.3390/ijms21103548 (PMC7278924; doi:10.3390/ijms21103548)

Figure S1a. ^{1}H NMR spectra of HPPTCA

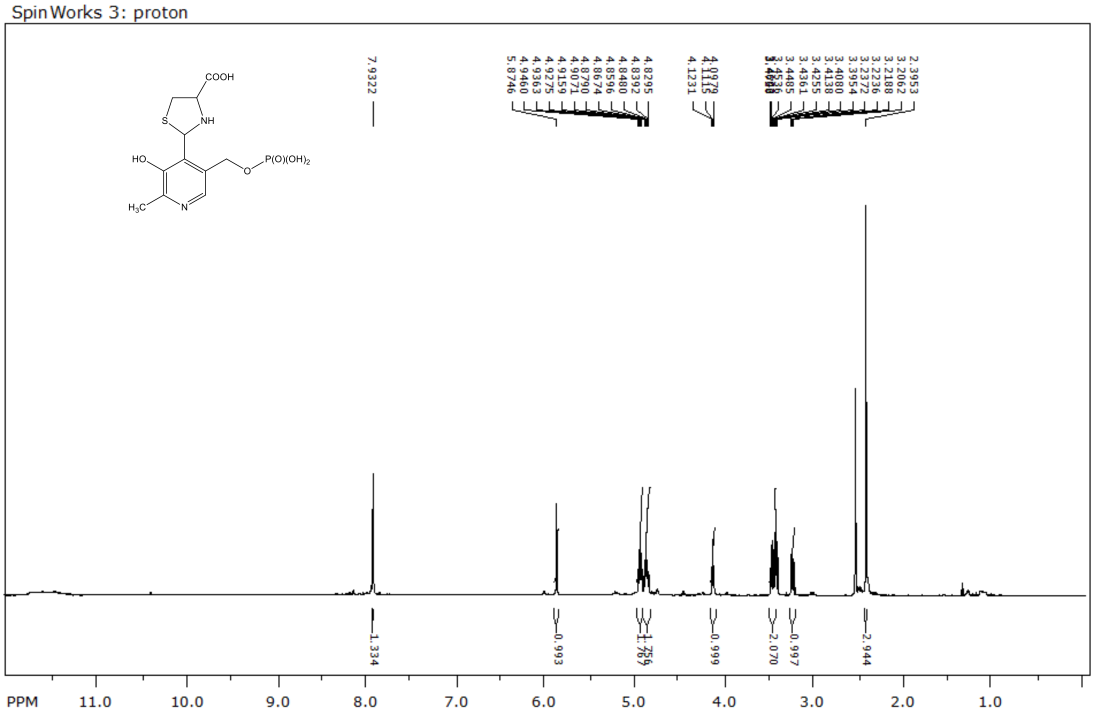

file: ...sers\48668\Desktop\widmo-ieg\2\fid expt: <zg30>
transmitter freq.: 600.263707 MHz
time domain size: 65536 points
width: 12335.53 Hz = 20.5502 ppm = 0.188225 Hz/pt
number of scans: 64

freq. of 0 ppm: 600.260000 MHz
processed size: 32768 complex points
LB: 0.000 GF: 0.0000
Hz/cm: 291.159 ppm/cm: 0.48505

Figure S1b. ^{13}C NMR spectra of HPPTCA

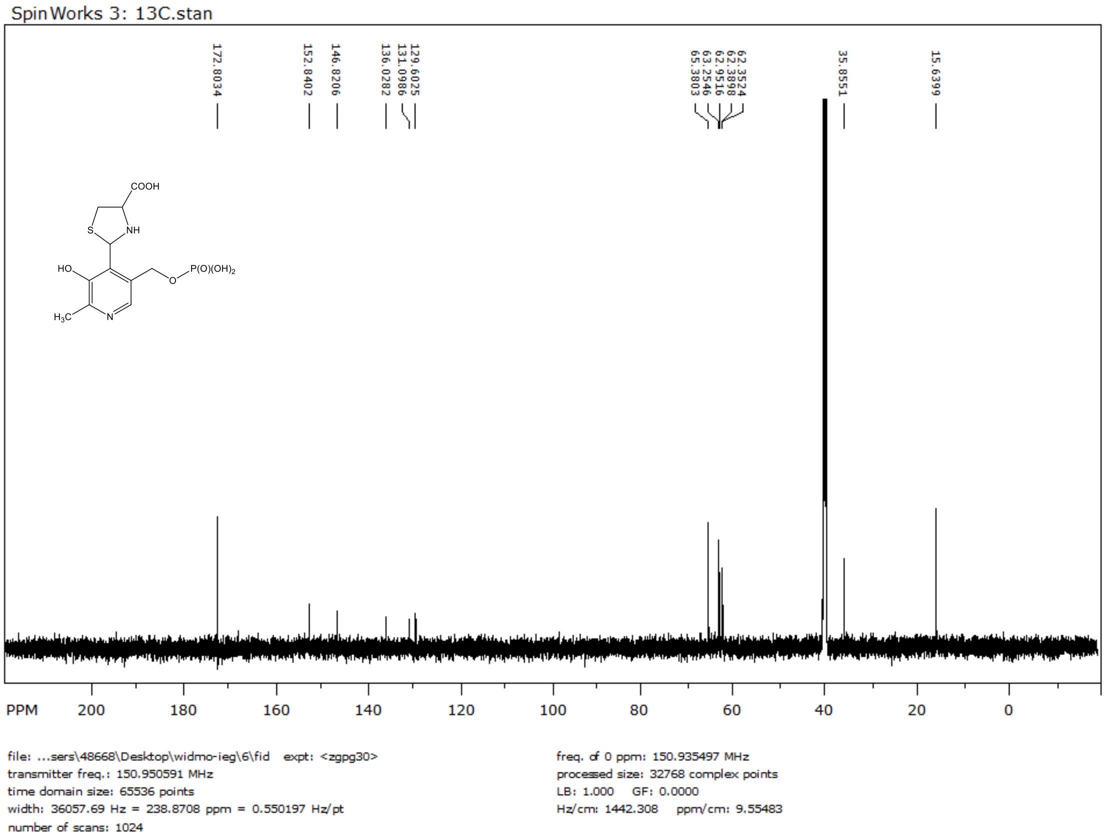

Figure S1c. ^{31}P NMR spectra of HPPTCA

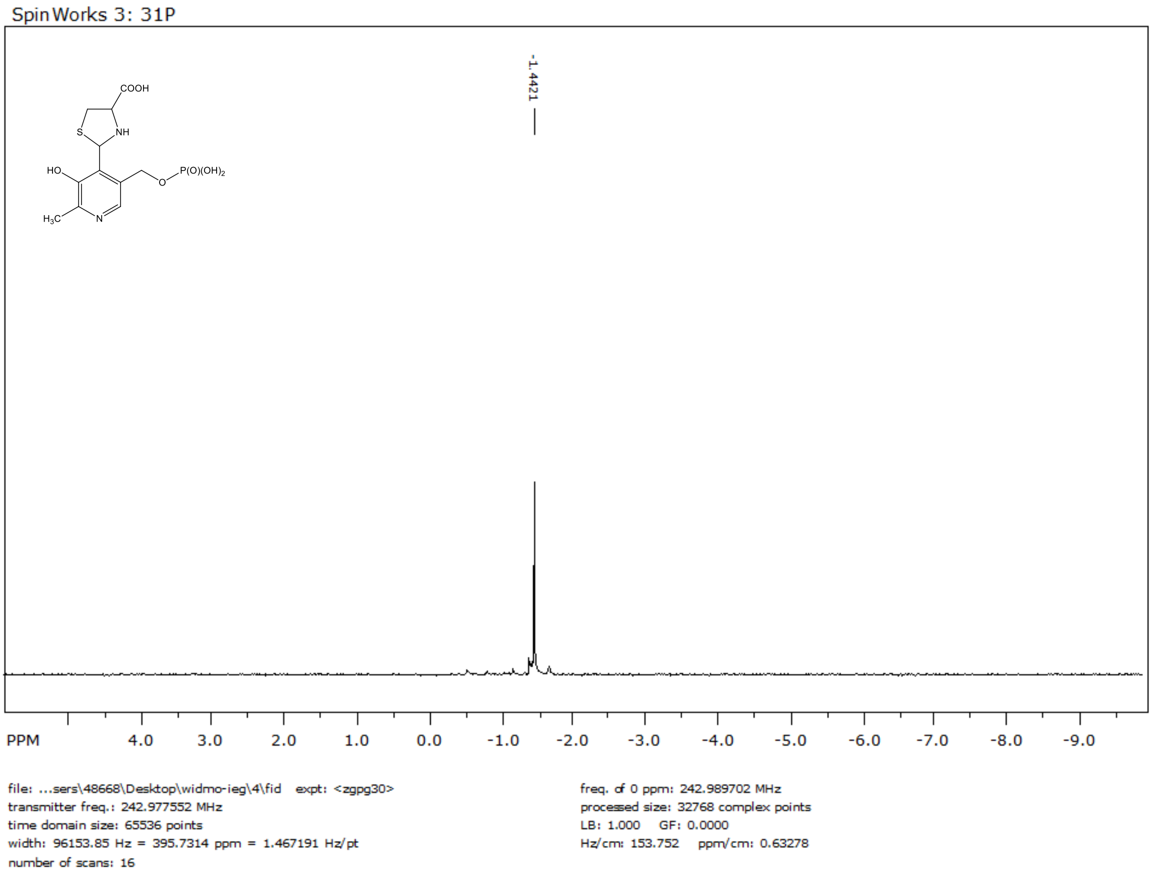

Figure S2. Protonated [M+H]⁺ at *m/z* 351.00 and deprotonated [M-H] at *m/z* 349.05 molecular ions of HPPTCA obtained in positive and negative mode, respectively.

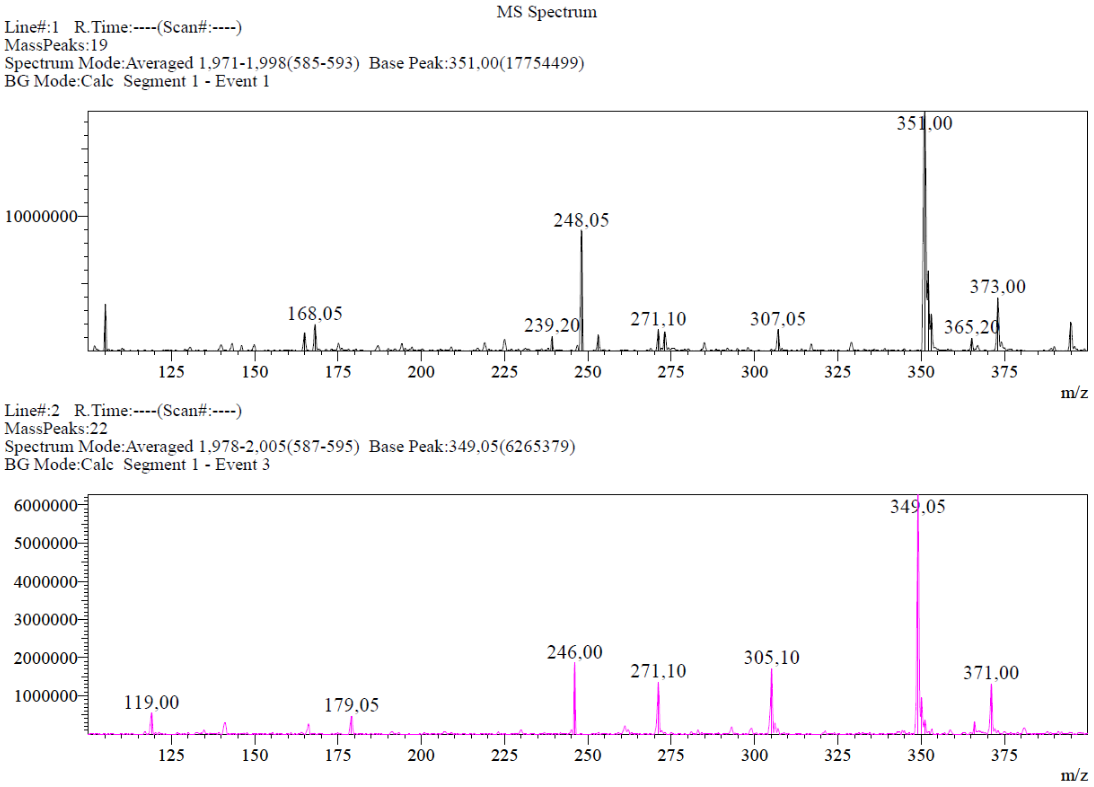

Supplement: Supplementary file 1 [file ijms-21-03548-s001.pdf]
